# A Prospective Evaluation of Serum Vitamin D (1, 25(OH)_2_ D_3_) and Endogenous Sex Hormone Levels in Colorectal Cancer Patients

**DOI:** 10.3389/fonc.2019.00468

**Published:** 2019-06-04

**Authors:** Suhail Razak, Iftikhar Alam, Tayyaba Afsar, Mahmoud M. A. Abulmeaty, Ali Almajwal, Sarwat Jahan

**Affiliations:** ^1^Department of Community Health Sciences, College of Applied Medical Sciences, King Saud University, Riyadh, Saudi Arabia; ^2^Department of Animal Sciences, Quaid-i-Azam University, Islamabad, Pakistan

**Keywords:** colorectal cancer, vitamin D (1, 25(OH)_2_ D_3_), sex hormones, gender comparison, univariate analysis

## Abstract

**Background:** Data on 25-OH VD concentrations and the associated factors in colorectal cancer (CRC) patients are scarce and need to be investigated.

**Methods:** A total of 200 CRC patients participated in this cross-sectional study conducted in Pakistan. Socio-demographic and other health data were collected in a pretested questionnaire. Serum measurements of Vitamin D (1, 25(OH)_2_ D_3_) levels and hormones were performed. Association of age, sex, primary site, effects of hormone therapy and stage of disease and selected reproductive health indicators on vitamin D status were primarily scrutinized by univariate analysis.

**Results:** Mean age of the population was 55.3 years (±15.6; Range: 20–90 years). Estradiol concentration was considerably elevated in young females compared to young male patients (*p* < 0.001). The concentrations of FSH, LH testosterone and estradiol were significantly lower in post-menopausal female CRC patients as compared to their male counterparts of old age (*p*, for all trends < 0.05). Both LH and FSH showed significant gender difference but only in older patients. Level of estrogen was markedly decreased in older post-menopausal CRC patients compared to premenopausal CRC patients, which might be associated with CRC progression. In the group of women, who “ever used hormone therapy” had differences of statistical significance (*p*, for all trends < 0.05) in their mean serum 25-OH VD concentrations, while in the group of women who “never used hormone therapy” had non-significant differences in their mean serum 25-OH VD concentrations (*p*, for all trends > 0.05). High 25-OH VD concentrations were observed in women who had their menarche at the age of 15 years or more. Nulliparous women had the highest mean 25-OH VD concentrations as compared to unparious or multiparious women. In addition, women having their menopause at 40–44 years of age had the highest 25-OH VD concentrations, although the difference was not significant (*p* = 0.08). Women who “never used any oral contraceptive” had higher 25-OH VD concentrations as compared to those “whoever used oral contraceptives.”

**Conclusion:** Our findings suggest that vitamin D has a positive effect on the development of CRC through the mediation of hormones. Other health and reproductive traits that affect hormone levels may have an indirect effect on the development of CRC. Further potential studies that directly evaluate levels of circulating hormones and hormone therapy in women in association to 25-OH VD concentrations, as well as their possible role in colorectal cancer risk, would be vastly edifying.

## Background

The worldwide prevalence of colorectal cancer (CRC) is third among cancer rate of recurrence in men and fourth among women (1, 2). In addition, colonic adenocarcinoma accounts for 37–45% of all metastatic ovarian tumors (3). Even though there is a decline in the death rates for colorectal cancers from 1990 to the present and even with advances in screening and surgical treatment, no cure has been discovered for metastatic cancer and the 5–6-year survival rate is regretfully low (almost 7.8%) (4, 5).

Men are predisposed to have a higher incidence of colorectal cancer than women of the same age (1, 2). In families in the midst of hereditary non-polyposis colorectal cancer (HNPCC), the survival risk of mounting progression of colon cancer to a great extent inferior in females (37%) than in men (78%) (6). The determination of the sex differentiation over decades, in various racial traits and all over the world revealed that sex hormones of both males and females play a vital role in the pathogenesis of CRC and their roles are comparatively defensive (7, 8). Numerous hypotheses regarding the relationship of reproductive factors and use of exogenous sex hormones and progression of colon cancer have been strengthened by results from observational studies (9). Various research analyses have found a reduced risk of colon or CRC in relation to “ever” vs. “never” use of menopausal hormones (7, 8). Murphy et al. found that endogenous revelations to sex hormones didn't reveal significant relation with CRC risk (10). On the other hand, the shielding function of the estrogen pathway in the progression of colon cancer has been reported in various animal models. For example, estrogen therapy has been shown to slow down colon tumors development in rats (11–13). Estrogens role in CRC is under consideration with bottomless attention particularly in context to the patho-physiology of cancers of endometrium, prostate, breast, colon, and rectum (14). The accurate mechanism of function of estrogen in CRC till date remains a controversial topic (15). Nevertheless, endogenous estradiol has been shown to have a vital role in the genetic pathway of CRC and the circulating levels of estradiol are associated with an elevated possibility of cancer (16). Estrogen prejudices the mucosal response to inflammatory injury in colitis, hence encourages inflammation-associated cancer progression (17). A comprehensive and relatively large-scale meta-analysis by Grodstein et al. revealed a positive association between hormone replacement therapy (HRT) and declined risk of colon cancer as much as of approximately 34% (18). The association was further strengthened by the Women's Health Initiative (WHI) Clinical Trial (19, 20), where involvement with estrogen added progestin results a 45% decrease in CRC, whilst only estrogens showed no concerning CRC risk. As epidemiological data support a shielding consequence of HRT on CRC, the links between different amalgamations of HRT and CRC danger remain unrevealed. Very few studies revealed the alliance among sex hormone intensities and colorectal cancer risk in males. Similar to the controversial role of estrogens in females, studies on the association of testosterone concentration and CRC progression are divisive. Studies have hypothesized that lower androgenicity may boost men's risk of mounting CRC probably during the initiation of androgen receptor signaling pathway (21). This advocates that males with lower androgenicity because of diminished AR activity or low circulating androgens were at larger jeopardy of CRC. Whereas, recent studies have revealed, that testosterone increase likelihood of developing CRC in men. Such observations were strengthened by animal studies which showed that orchidectomised mice have a minor risk of mounting CRC than controls. These observations proposed that testosterone may encourage colorectal adenoma formation, resulting in higher male susceptibility to CRC. Besides that, female mice with ovarectomy illustrated the greater risk of mounting CRC when compared with female mice without ovarectomy (22). These observations sustain the impression that sex hormones may confer defense against CRC or encourage the progression of CRC as the case with testosterone. Consequently, quantification of serum testosterone and other sex hormones could be an attractive prospective predictive biomarker of CRC risk. Clinically serum testosterone is used as a biomarker for prostate cancer. Also has been anticipated as a means for quantifying prognosis of breast cancer in postmenopausal women. Nevertheless, data of its use in CRC is limited and promising facts on the role which testosterone plays in CRC warrants auxiliary long-term clinical trials.

The substantial established molecular and genetic data advocate a role of vitamin D against CRC. In addition, many epidemiological studies identified that vitamin D insufficiency is associated with elevated risk of this neoplasia. Worldwide the results accessible on the loss of VDR expression and on the amendment of CYP27B1 and CYP24A1 levels at some stage in CRC development, sustain a role for vitamin D in the preclusion and/or in the therapy of initial stages rather than in the treatment of advanced cases of this neoplasia (23–27). Numerous studies have revealed an association between CRC and 25-OH vitamin D deficiency (28–30). Regardless of the involvement between vitamin D status and jeopardy of colorectal cancer, inadequate data subsists on vitamin D and CRC development across stages I–IV. In addition, veto data exist on vitamin D status and hormones levels in colorectal cancer. A study revealed that 25-OH vitamin D levels in colon cancer cases were higher than the controls. In addition, 25-OH D3 levels showed consistency across the various stages of colorectal cancer, while 1, 25-OH vitamin D diminished with mounting stage (31).

Given the limited data on vitamin D status and association of sex hormones across various stages of CRC and in view of promising clinical data sustaining an impact of vitamin D status on cancer outcome, we examined vitamin D and sex hormones status in 200 CRC patients. Our main objective was to investigate any possible association between sex hormones and vitamin D levels at different stages of the disease in a less studied population of Pakistan.

## Methods

The study was carried out at Reproductive Physiology Laboratory, Department of Animal Sciences Quaid-i-Azam University, Islamabad, Pakistan from December 2016 to November 2017. The study was approved by the Institutional Review Board (IRB) of Quaid-i-Azam University, Islamabad, Pakistan. All participants were informed about the study objectives and signed informed consent. The study protocol was carried out in accordance to the principles of the Declaration of Helsinki (32).

### Selection of Case Participants and Collection of Blood Samples

Cases of CRC patients were newly diagnosed, histopathologically confirmed and diagnosed with adenocarcinoma of the colon and/or rectum.

Venous blood samples from all patients were available from the Department of Urology, AFIP, Rawalpindi, Pakistan. At the time of blood sampling, the age of patients ranges from 32 to78 years. In female CRC patients, data on reproductive and menstrual health included data age at menarche, use of hormonal contraceptives, menopausal status, the cause of regularity of the menstrual cycle, menopause, and hormone replacement therapy (HRT). Other details of reproductive history included a number of miscarriages, number/order/sex of the children, gestational age and duration of maternal lactation and information on fertility problems and their treatment was collected. Data on demographic and clinical features were taken and included gender, age at the time of diagnosis, family history, cell type, disease localization, grade, and stage of the tumor (Table 1).

**Table 1:** Baseline characteristics of CRC patients (*n* = 200).

**Sex**	**Number of patients**	**Average age**	**Locatization**	**Cell type**	**Grade**	**Stage**	**Smoker (S) non smokers (NS)**	**HRT therapy yes/no**	**Family history of CRC**
Males (M)	136	53.67	Rectum	Adenocarcinoma	Well Differentiated	T0 NO MO	60 (S)	No	130 no
			28(M)	100(M)	106(M)	T1 N1 Mx	76(NS)		6 yes
			08(F)	58(F)	60(F)				
Females (F)	64	56.03	Colon	Mucinous Carcinoma	Moderately Differentiated	T2 NO Mx	4 (S)	22 yes	2 yes
			92(M)	30(M)	27(M)	T2 N1 Mx	60(NS)	42 no	62 no
			49(F)	04(F)	03(F)	T2 Nx Mx			
			Recto	Signet Ring cell	Poorly	T3 NO MO			
			sigmoid	carcinoma	Differentiated	T3 N0 M1			
			17(M)	06(M)	03(M)	T3 N0 Mx			
			04(F)	02(F)	01(F)	T3 N1 Mx			
			Cacum			T3 N2 MO			
			09(M)			T3 N2 M1			
			03(F)			T3N2Mx			
						T4 N0 Mx			
						T4 N1 M1			
						T4 N2 Mx			
						T4 N3 M1			

## Laboratory Methods

### Estimation of Circulating Sex Factors

Serum levels of LH, testosterone, and estradiol representing female and male sex hormones were calculated by using commercially available enzyme-linked immunosorbent assay (ELISA) kits (Phoenix Pharmaceuticals, INC, USA), according to manufacturer's instructions.

### Measurement of Vitamin D

Serum level of Vitamin D (1, 25(OH)_2_ D_3_) was measured by ELISA kit (MyBioSource) following standard manufacturer protocol.

### Statistical Analysis

Univariate analysis was used to assess the effect of sex, age, primary site, and stage of disease on vitamin D status. The distribution pattern of sex hormones and 25-OH vitamin D levels in relation to the stage of CRC in young and old CRC patients were evaluated. Pearson's correlation analyses were performed to see the association of 25-OH vitamin D with the stage of the disease in both premenopausal and postmenopausal females with CRC. Odds ratio (OR) was calculated considering ≤16 ng/ml) 25-OH vitamin status as “very low” with respect to the default or reference level (33). To simultaneously consider the impact of age, sex, primary site and stage of disease on 25-OH vitamin status, multiple logistic regression analyses were performed by fitting the log of odds (with “very low” 25-OH vitamin status) on the explanatory variables. We also calculated Hazard Ratio (HR) as described previously. We stratified the data of female patients by hormone therapy use to observe any significant interaction between the mean age of female patients, use of hormone therapy, and serum 25-OH VD status. Statistical significance tests for trend (P-trend) and interaction (P-interaction) were calculated. All data were expressed as mean (STD). An alpha value (*p*-value) was considered significant at 0.05.

## Results

### Demographics of Male and Female Patients

Table 1 indicates the demographic characteristics of CRC patients. The percentage number of rectal and colon patients were 20% and 80, respectively. Mean age of the patients 55.3 years (±15.6; Range: 20–90 years). All patients were of normal body weight (mean BMI = 23.5; Range 20–24.8). Mean (SD) 25-OH vitamin D levels was 18.8 (9.11) ng/ml.

### Sex Hormones Concentrations in CRC Patients

Table 2 shows serum concentrations of testosterone, estradiol, FSH, and LH hormones in young male vs. young female. Only estradiol concentration was considerably elevated in young female than young male patients (*p* < 0.001). Table 2 also shows serum concentrations of testosterone, estradiol, FSH, and LH hormones in old male vs. post-menopausal female CRC patients. As evident, the concentrations of these hormones were significantly lower in post-menopausal female CRC patients as compared to their male counterparts of old age (*p*, for all trends < 0.05).

**Table 2:** Sex hormone concentrations of CRC patients.

	**Males**	**Females**	***P*-value**
**A. HORMONE CONCENTRATIONS IN YOUNG MALE AND PRE-MENOPAUSE FEMALE**			
Mean age (years) (*n* = M/F = 38/25)	38.87 ± 7.27	35.60 ± 8.92	0.116
Testosterone (ng/dl)	1572.25 ± 262.58	102.08 ± 3.72	<0.001^**^
Estradiol (pg/ml)	42.52 ± 16.66	50.51 ± 23.58	0.120
FSH (mlU/ml)	31.96 ± 4.52	31.02 ± 3.86	0.394
LH (mlU/ml)	33.09 ± 4.64	33.13 ± 2.96	0.969
**B. HORMONE CONCENTRATIONS IN OLD MALE AND POST-MENOPAUSE FEMALE**			
Age (years) (*n* = M/F = 74/47)	64.47 ± 8.92	64.60 ± 10.66	0.946
Testosterone (ng/dl)	1648.25 ± 270.23	105.27 ± 7.18	<0.001^**^
Estradiol (pg/ml)	49.72 ± 10.91	16.86 ± 5.30	<0.001^**^
FSH (mlU/ml)	32.18 ± 4.53	34.42 ± 3.05	0.003^*^
LH (mlU/ml)	31.84 ± 4.55	34.17 ± 3.49	0.003^*^

* Denotes statistical significant differences at an alpha value (p-value) of ≤0.05 using students t test;

** *Indicates significant difference at an alpha value (p-value) of <0.0001 between Pre-menopause female and Post-menopause female using students t test*.

In postmenopausal cases, physiological reduction of estradiol level makes a significant difference (*P* < 0.001) in estradiol level between males and females with high levels in males of the equivalent ages. Both LH and FSH showed significant gender difference only in older age. Furthermore, the comparison of hormone concentration between premenopausal and post-menopausal females revealed that the level of estrogen is markedly decreased in older post-menopausal CRC patients, which might be associated with CRC progression.

### Impact of Patients Demographics on Vitamin D Status

Table 3 shows the distribution of 25-hydroxyvitamin D [25(OH) D] levels in CRC patients. For the current observation, 25-OH VD status was classified into two groups: “very low” and “low to normal.” The “very low” group was defined as ≤16 ng/ml and the “low to normal” group was defined at >16 ng/ml. Levels below 16 ng/ml have been traditionally considered as low. In addition, ≤16 ng/ml corresponds to the lowest quartile of our population. Variables investigated in this study included age, sex, primary site (colon vs. rectum), stage of disease (stages I–III vs. IV). BMI and date of 25-OH vitamin D assay were not considered for regression analysis as all patients had normal BMI (Range 22–24.9) and blood was collected from all patients in the same month of the year thus excluding the possibility of significant seasonal variations in serum vitamin D concentrations (Table 4).

**Table 3:** 25-hydroxyvitamin D [25(OH) D] status in CRC patients.

**Parameters**	**Vitamin D**	***P*-value**
**Mean age**		
<50 years	22.1 (9.19)	0.032^*^
>50 years	18.8 (9.1)	
**Gender**		
Male	17.1 (9.3)	0.021^*^
Female	21.5 (8.6)	
**Site of cancer**		
Rectum	22.1 (7.9)	0.012^*^
Colon	14.8 (5.7)	
**Stage of disease**		
Stage I–III	19.6 (9.8)	0.023^*^
Stage IV	16.4 (5.6)	

* *Denotes statistical significant differences at an alpha value (p-value) of ≤0.05 using students t test*.

**Table 4:** Univariate and multivariate logistic regression analysis of low vs. normal 25-OH VD level (≤15 vs. >15 ng/ml).

**Category**	**Univariate analysis OR (95% CI)**	***p*-value**	**Multivariate analysis OR (95% CI)**	***p*-value**
Age (<50 vs.>50years)	2.42 (1.29–4.55)	0.004^*^	1.78 (0.87–3.66)	0.110
Gender (female vs. male)	1.87 (0.98–3.42)	0.038^*^	1.97 (1.023–3.82)	0.142
Site of cancer (rectum vs. colon)	1.67 (0.87–3.21)	0.031^*^	1.24 (1.13–3.45)	0.150
Stage (stage I, II, III vs. IV)	2.44 (1.22–4.91)	0.012^*^	1.94 (1.07–3.56)	0.031^*^

* *Denotes statistical significant differences at an alpha value (p-value) of ≤0.05 using students t test. OR, odds ratio; CI, confidence interval*.

### Reproductive Factors and Distribution of 25-OH VD Concentrations in CRC Patients

Table 5 shows data on 25-OH VD concentration in female patients according to various reproductive factors. As shown, female patients having their menarche before or after the age of 10–12 years of age had significantly lower 25-OH VD level (*p* = 0.02). Similarly, the nulliparous female had significantly higher 25-OH VD levels as compared to those having children (*p* = 0.04).

**Table 5:** Reproductive factors and 25-OH VD concentration in female CRC patients (*n* = 54).

**Reproductive factors**	**25-OH VD level (ng/ml)**	***p*-value**
**Menarche age**		
<10	13.7 (6.4)	0.02^*^
10–12	18.4 (5.2)	
12–14	20.6 (5.5)	
>15	18.6 (6.5)	
**Parity**		
Nulliparous	21.5 (5.4)	0.04^*^
1 child	19.5 (5.4)	
2 children	18.3 (5.4)	
3 children	15.4 (5.4)	
>4 children	13.8 (4.4)	
**Menopause age**		
<40	20.3 (5.9)	0.09
40–44	20.8 (8.3)	
45–49	18.1 (6.4)	
50–54	18.2 (7.2)	
>55	16.1 (8.5)	
**Oral contraceptive use**		
Never	21.3(5.1)	0.02^*^
Ever	16.2 (3.7)	

* *Denotes statistical significant differences at an alpha value (p-value) of ≤0.05 using students t test*.

Women with menopause at age <40 years had higher 25-OH VD level as compared to women who had their menopause after the age of 40. However, this difference didn't reach statistical significance (*p* = 0.09). Women who ‘never used oral contraceptive’ had higher 25-OH VD levels as compared to those who ‘ever used oral contraceptives’ (*p* = 0.02).

In the auxiliary investigation, we stratified the data of female patients by hormone therapy use (Table 6) and observed statistically significant differences in serum 25-OH VD concentrations of female patients because of differences in their reproductive health characteristics and use of hormone therapy. In general, the “ever used hormone therapy” female group had differences of statistical significance (*p*, for all trends < 0.05) in their mean serum 25-OH VD concentrations, while in the group of females who “never used hormone therapy” had non-significant differences in their mean serum 25-OH VD concentrations (*p*, for all trends > 0.05). Furthermore, taken as single groups (ever use hormone therapy vs. never use of hormone therapy) women with “ever use of hormone therapy” had significantly higher 25-OH VD concentrations as compared to women with “never use of hormone therapy” (*p*, for all trends < 0.05).

**Table 6:** Serum 25-OH VD concentrations according to reproductive and menstrual factors by hormone therapy (*n* = 54).

**Factor**	**Ever use of hormone therapy (*n* = 29)**	**Never use of hormone therapy (*n* = 25)**	***p*-value**
**Mean age**			
<50 years	22.5 (9.19)	19.5 (9.19)	0.002^*^
>50 years	19.8 (9.1)	18.8 (8.1)	
	*P* = 0.02	*P* = 0.24	
**Menarche age**			
<10	14.2 (5.8)	13.2 (5.3)	0.04^*^
10–12	21.4 (5.3)	15.4 (6.3)	
12–14	26.8 (3.8)	14.4 (7.7)	
>15	22.4 (8.7)	14.8 (3.9)	
	*P* = 0.01	*P* = 0.26	
**Parity**			
Nulliparous	26.4 (6.8)	16.4 (3.9)	0.04^*^
1 child	23.2 (4.7)	15.4 (6.9)	
2 children	21.3 (4.9)	15.2 (7.1)	
3 children	15.3 (4.2)	15.5 (3.9)	
>4 children	14.6 (6.8)	13.1 (2.9)	
	*P* = 0.02	*P* = 0.32	
**Menopause age**			
<40	21.3 (5.9)	19.4 (6.5)	0.03^*^
40–44	23.3 (12.4)	18.4 (4.5)	
45–49	18.7 (8.7)	18.3(4.9)	
50–54	18.3 (9.3)	17.9 (5.9)	
>55	16.2 (7.3)	16.8 (9.7)	
	*P* = 0.08	*P* = 0.09	
**Oral contraceptive use**			
Never	26.1 (2.8)	17.3 (9.8)	0.04^*^
Ever	16.5 (3.9)	15.8 (4.8)	
	*P* = 0.02	*P* = 0.21	

* *Denotes statistical significant differences at an alpha value (p-value) of ≤0.05 using students t test*.

**Figure 1:**
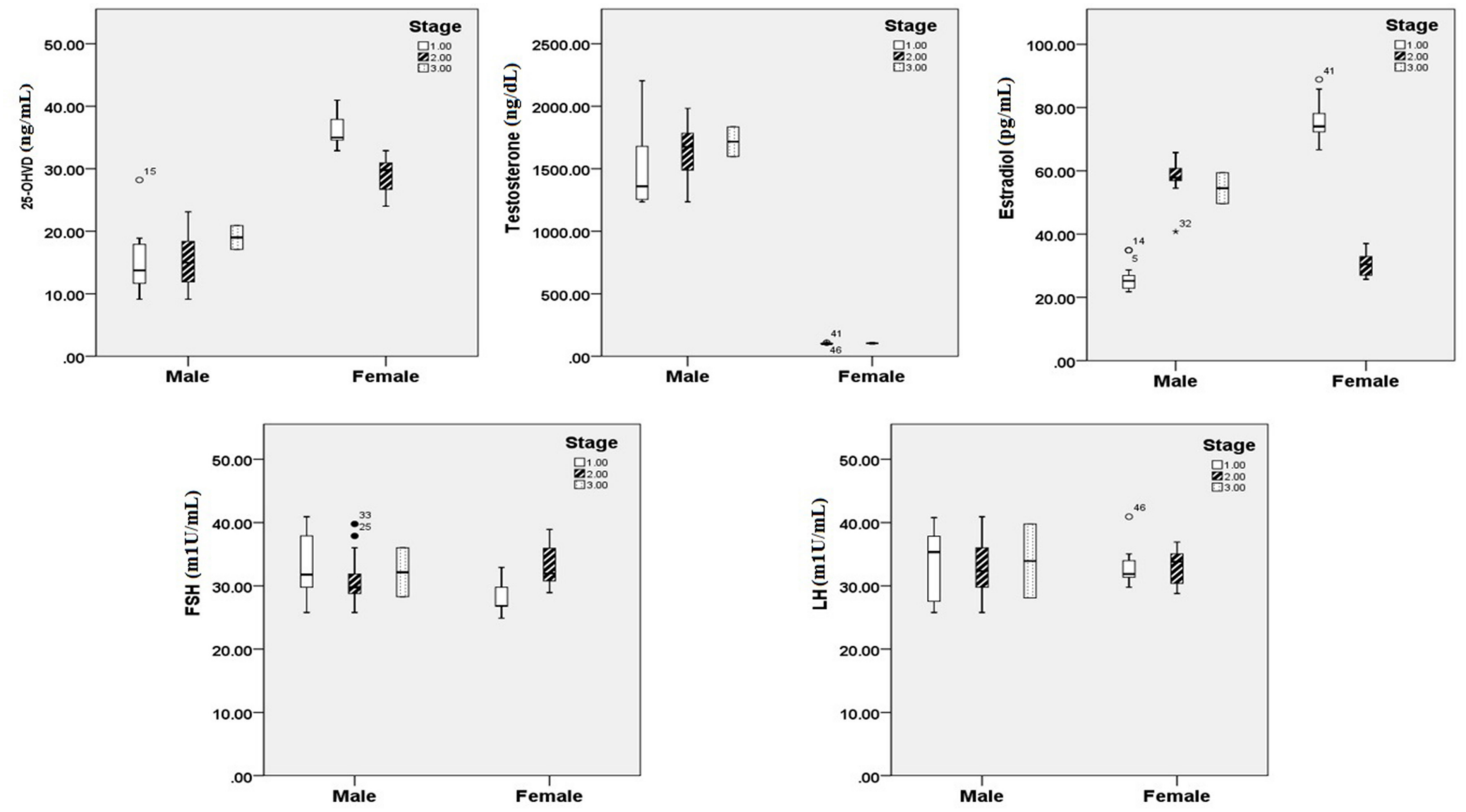
25 OH vitamin D and sex hormonal patterns in relation to the stage of CRC in young participants (*n* = 200). Pearson's correlation at alpha = 0.05.

**Figure 2:**
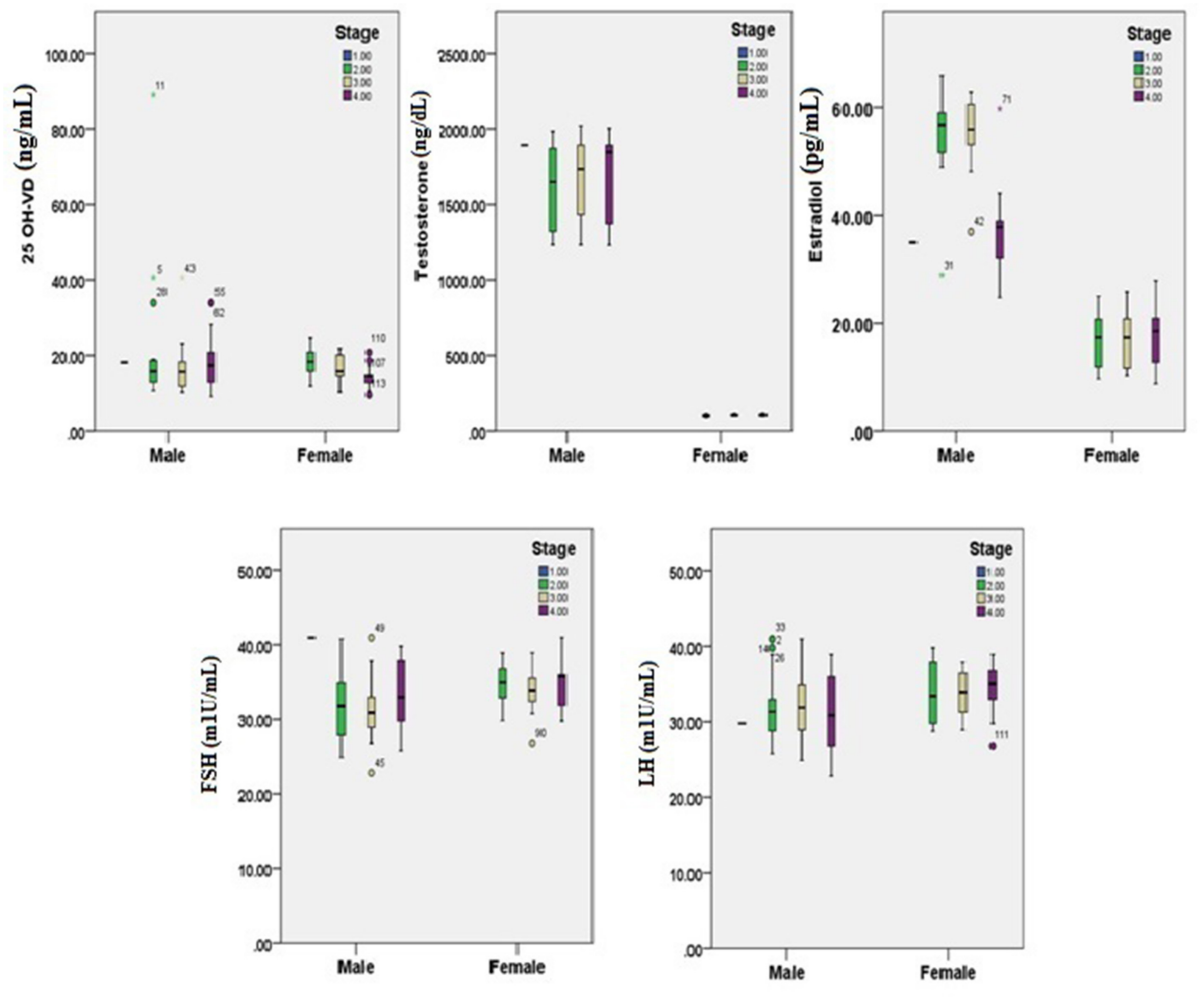
25-OH vitamin D and sex hormones patterns in relation to the stage of CRC in old participants (*n* = 200). Pearson's correlation at alpha = 0.05.

Figures 1, 2 reveals the analyzed distribution pattern of sex hormones and 25-OH vitamin D levels in relation to the stage of CRC in young and old CRC patients. In younger female cases, early stages have relatively higher levels of 25-OH vitamin D and estradiol which get reduced with the progression of the CRC. Furthermore, 25-OH vitamin D showed strong negative correlations with the stage of the disease in both premenopausal and postmenopausal females with CRC (*r* = −0.808 and −0.434, respectively, *P* < 0.01, Table 7). This was not the case in males in both age groups. Regarding estradiol level in young males, there was a strong positive correlation with the stage of the disease (*r* = 0.877, *p* < 0.01), while in older ages the correlation became negative (*r* = −0.643, *p* < 0.01). Females in the reproductive ages and with CRC showed an extremely strong negative correlation with the stage of the disease (*r* = −0.976, *p* < 0.01). In both genders and age groups, the severity of the disease stage correlated positively with the age.

**Table 7:** Correlation (*r* values) of various investigated parameters with the stage of CRC.

**Parameters**	**Males**		**Females**	
	**Young, <50 years (*n* = 38)**	**Old, >50 years (*n* = 74)**	**Premenopausal (*n* = 25)**	**Postmenopausal (*n* = 47)**
Age	0.627^**^	0.571^**^	0.817^**^	0.0764^**^
VD	0.163	−0.013	−0.808^**^	−0.434^**^
Testosterone	0.297	0.103	0.229	0.282
Estradiol	0.877^**^	−0.643^**^	−0.976^**^	0.014
FSH	−0.183	0.045	0.686^**^	0.001
LH	−0.013	0.030	0.045	0.063

** Correlation is significant at the 0.01 level (2-tailed); *Correlation is significant at the 0.05 level (2-tailed).

## Discussion

The present study assessed serum 25-OH Vitamin D concentrations of 200 colorectal cancer (CRC) patients. The sample comprised of 122 male and 78 female with mean age of 55.8 ± 6.9 and 54.5 ± 14.6 years, respectively. The present study report main three findings:

### Age and Gender as a Determinant of Hormone Levels

When subjects were divided into young males and female groups, no significant difference was observed in the serum concentrations of hormone levels (*p*, for all trends > 0.05; Table 2). However, significant differences were observed in the serum concentrations of old male and female (*p*, for all trends < 0.05; Table 2).

### Age, Gender, Site of Cancer, as Determines of Serum 25-OH Vitamin D Concentrations

Findings of the present study show that mean 25-OH vitamin D levels in the patients were 18.8 ± 9.11 ng/ml. 25-OH VD status was classified into two groups “very low” and “low to normal.” The “very low” category was defined as ≤16 ng/ml and the “low to normal” category was defined at >16 ng/ml (34). The other reason for selecting this threshold was that 25-OH VD levels of our subjects were within these ranges. Regression analysis using univariate model show age, gender, site of cancer, and stage of disease were found to be associated with the disease. Age, gender, site of cancer, and stage of disease were found to be 2.42, 1.87, 1.67, and 2.44 times, more likely to be allied with low 25-OH VD (*p*, for all trends < 0.05; Table 3). However, multivariate logistic regression results show that only stage of disease (OR: 1.94; CI: 1.07–3.56) was significantly associated with low 25-OH VD (*p* = 0.031; Table 4).

### There Is a Close Association Between Serum 25 (OH) D Level and Sex Factors

Early reports have illustrated that amplified levels of testosterone and estrogen, in males and females, are certainly related with serum 25 (OH) D levels (33, 35, 36). We, therefore, analyzed the distribution pattern of sex hormones and 25-OH vitamin D levels in relation to the stage of CRC (Figures 1, 2). Estrogen directly standardizes hepatic hepcidin expression through an efficient estrogen response factor in the promoter region of the hepcidin gene (37). In pre-menopausal women, in particular, 17β-estradiol amplifies iron uptake to reimburse for iron loss during menstruation (38). Nevertheless, postmenopausal women have an accelerated diminution of estrogens caused by menopause. Consequently, in spite of the increase in serum 25 (OH) D concentrations, the ferritin level may be not appreciably dissimilar in postmenopausal women. However, a study proposed that testosterone amplifies ferritin by bracketing hepcidin in men (39).

### Effects of Reproductive Characteristics on 25-OH VD Concentrations

We also analyzed some selected reproductive factors as predictors of 25-OH VD concentrations (Table 6). High 25-OH VD concentrations were observed in women who had their menarche at the age of 15 years or more. Nulliparous women had the highest mean 25-OH VD concentrations as compared to unparious or multiparious women. Women having their menopause at 40–44 years of age had the highest 25-OH VD concentrations, although the difference was not significant (*p* = 0.08). Women who never used any oral contraceptive had higher 25-OH VD concentrations as compared to those who ever used oral contraceptives. Furthermore, it is interesting that these differences were more profound when the women patients were stratified into two groups based on their hormone therapy i.e., ever use hormone therapy and never use hormone therapy (Table 6). So for example, women having menarche at age 15 years or more and who ever used hormone therapy had considerably higher 25-OH VD concentrations as compared to women who had menarche <15 years of age with either ever use hormone therapy or with never use hormone therapy (*p*, for all trends < 0.05) in the same way being nulliparous and parity (Table 6).

Early menarche is associated to elevated risk of poor health outcomes during adulthood including obesity (40), type 2 diabetes (41), cardiovascular disease (42), and breast (43) and endometrial cancers (44). Women with later onset of menarche demonstrate significantly decreased peak bone mass (45), and bone mass and bone density at skeletal maturity exhibit an inverse relationship with pubertal timing in healthy adolescents (46). Later age of menarche has also been associated with increased fracture risk (47). In this study, we observed mean lower 25-OH VD concentrations in women having their menarche age <10 or >15 years (Table 5). Like age at menopause, age at menarche is a marker of the period of exposure to cyclic ovarian function (48, 49). Studies have confirmed an inverse association between circulating estrogen level and age at menarche. Pregnant women and women breastfeeding for long periods of time are at higher risk of hypovitaminosis D (50–52). Later age at menopause is a recognized risk factor for the elevated number of ovulatory cycles and amplified estrogen exposure allied with later menopause has been theorized to constrain this association (53, 54). The mean age of menopause in Pakistan has been reported that varied greatly i.e., 44.5 years (55) to 47–49 years (56, 57). Similarly, the age of menarche in Pakistani women is also variable (58) and has profound health outcomes. These are important factors and may have significant effects on 25-OH VD concentrations and overall health of women both healthy and those suffering from chronic diseases like CRC.

### Limitations of Study

The present study has some limitations. Firstly, relatively small sample size did not allow us to carry out stratified analyses with abundant statistical power, which would have permitted us to estimate females according to the stage of disease and hormone therapy use; two factors that could have a perplexing effect on the alliance between reproductive history and 25-OH VD concentrations in colorectal cancer patients. Secondly, all of the prime variables of concern were based on self-reported reproductive history and therefore we cannot reject the possibility of bias associated to imprecise recall. However, self-reported reproductive history has shown good concurrence with medical records in validation studies (59, 60). Moreover, we could not further stratify the analyses by hormone therapy subtypes, and we lacked more detailed data on other reproductive factors, such as age at first pregnancy or age of mother at the birth of first and last child, breastfeeding history, and history of induced or spontaneous abortion, which have been linked with 25-OH VD concentrations (61–63) and which could help further elucidate the role of sex hormones in colorectal cancer development. Due to its cross-sectional nature, the present study is unable to conclude whether Vitamin D levels and hormonal factors are causative or merely correlative. Further case-control studies on a larger scale are needed to clarify this. Previous studies have shown the presence of 1, 25-(OH)_2_D_3_ receptors in some colorectal tumors (64). This needs further investigations into the possible role of 1, 25-(OH)_2_D_3_ on colorectal carcinogenesis. We, therefore, would recommend further prospective studies that directly assess levels of circulating hormones and hormone therapy in women in relation to 25-OH VD concentrations as well as their possible role in colorectal cancer risk at clinical as well as molecular and cellular levels.

## Conclusion

Our findings suggest that 25-OH VD has a positive effect on the development of CRC through the mediation of hormones. Other health and reproductive traits that affect hormone levels may have an indirect effect on the development of CRC. We suggest that studies in the future may evaluate levels of circulating hormones and hormone therapy in women in association to 25-OH VD concentrations as well as their possible role in colorectal cancer risk would be promising revealing.

## Ethics Statement

The study was approved by the Institutional Review Board (IRB) of Quaid-i-Azam University, Islamabad, Pakistan. All participants were informed about the study objectives and signed an informed consent. The study protocol was done in accordance with the principles of the Declaration of Helsinki.

## Author Contributions

SR designed the study, conceived the study and analyzed the results. IA, TA, and MA conceived an initial part of the study, performed the experiment, histology and helped in compiling the results. SR and TA performed experiment. AA helped in writing the results. SR and SJ wrote the paper with input from all other authors SJ, SR, TA, and AA made substantial contribution in interpretation of data and revising the manuscript for intellectual content. All authors read and approved the final manuscript.

### Conflict of Interest Statement

The authors declare that the research was conducted in the absence of any commercial or financial relationships that could be construed as a potential conflict of interest.

## Abbreviations

CRC: Colorectal Cancer
HNPCC: Hereditary non-polyposis colorectal cancer
LH: Luteinizing hormone
FSH: Follicle stimulating hormone
HRT: hormone replacement therapy
EDTA: Ethylenediaminetetraacetic acid
AR: Androgen Receptors
VDR: Vitamin D Receptor
HR: Hazrd Ratio.
