# Corrigendum: A Prospective Evaluation of Serum Vitamin D (1, 25(OH)_2_ D_3_) and Endogenous Sex Hormone Levels in Colorectal Cancer Patients

**DOI:** 10.3389/fonc.2020.00200

**Published:** 2020-02-25

**Authors:** Suhail Razak, Iftikhar Alam, Tayyaba Afsar, Mahmoud M. A. Abulmeaty, Ali Almajwal, Sarwat Jahan

**Affiliations:** ^1^Department of Community Health Sciences, College of Applied Medical Sciences, King Saud University, Riyadh, Saudi Arabia; ^2^Department of Animal Sciences, Quaid-i-Azam University, Islamabad, Pakistan

**Keywords:** colorectal cancer, vitamin D (1, 25(OH)_2_ D_3_), sex hormones, gender comparison, univariate analysis

In the published article, there was an error in the order of the affiliations. Affiliation 1 and 2 have now been switched with the correct order appearing above.

The authors apologize for this error and state that this does not change the scientific conclusions of the article in any way. The original article has been updated.

